# Degree of Agreement between Cardiovascular Risk Stratification Tools

**DOI:** 10.5935/abc.20170057

**Published:** 2017-05

**Authors:** Guilherme Thomé Garcia, Ana Maria Nunes de Faria Stamm, Ariel Córdova Rosa, Antônio Carlos Marasciulo, Rodrigo Conill Marasciulo, Cristian Battistella, Alexandre Augusto de Costa Remor

**Affiliations:** 1 Universidade Federal de Santa Catarina (UFSC), Florianópolis, SC - Brazil; 2 Hospital Universitário Prof. Dr. Polydoro Ernani São Thiago, Florianópolis, SC - Brazil

**Keywords:** Cardiovascular Diseases / mortality, Cardiovascular Diseases / morbidity, Risk Assessment, Cardiovascular Diseases / epidemiology, Period Analysis

## Abstract

**Background:**

Cardiovascular disease (CVD) is the leading cause of morbidity and mortality in Brazil, and primary prevention care may be guided by risk stratification tools. The Framingham (FRS) and QRISK-2 (QRS) risk scores estimate 10-year overall cardiovascular risk in asymptomatic individuals, but the instrument of choice may lead to different therapeutic strategies.

**Objective:**

To evaluate the degree of agreement between FRS and QRS in 10-year overall cardiovascular risk stratification in disease-free individuals.

**Methods:**

Cross-sectional, observational, descriptive and analytical study in a convenience sample of 74 individuals attending the outpatient care service of a university hospital in Brazil between January 2014 and January 2015. After application of FRS and QRS, patients were classified in low/moderate risk (< 20%) or high risk (≥ 20%).

**Results:**

The proportion of individuals classified as at high risk was higher in FRS than in QRS (33.7% vs 21.6%). A synergic effect of male gender with systemic arterial hypertension was observed in both tools, and with for geriatric age group in QRS (p < 0.05) in high-risk stratum. The Kappa index was 0.519 (95%CI = 0.386-0.652; p < 0.001) between both instruments.

**Conclusion:**

There was a moderate agreement between FRS and QRS in estimating 10-year overall cardiovascular risk. The risk scores used in this study can identify synergism between variables, and their behavior is influenced by the population in which it was derived. It is important to recognize the need for calibrating risk scores for the Brazilian population.

## Introduction

Cardiovascular disease (CVD) is the main cause of mortality and morbidity in Brazil. It accounts for 20% of deaths in individuals over the age of 30 years, and its high prevalence is associated with inadequate control of risk factors. Identification of asymptomatic subjects at higher risk for CVD is a crucial step in public health policies, since effective control of these factors can reduce the mortality rate by up to 44%.^1,2^

The presence of risk factors allows identifying individuals at higher cardiovascular risk (CVR). Nonetheless, intuitive estimates may either overestimate the low risk or underestimate the high risk, for not considering the interaction between them.^3^

A variety of scores have been developed for CVR stratification, such as the Framingham (FRC) and QRISK-2 (QRS) score, which estimate overall CVR in ten years. FRC was improved in 2008 m and has been widely used in Brazil and in the world along with aggravating factors. QRS was created in the UK, and has been used for cardiovascular prevention in primary care.^4,5^

Despite its practicality and widespread use, FRS may underestimate or overestimate the risk in Hispanic and European populations. This fact motivated the development of QRS. However, studies have pointed out disagreements between both scores in estimating high risk, which may lead to the use of different therapies for the same patient.^6^

Considering both the importance of identifying asymptomatic patients at high risk for CVD, and therapeutic implications of each risk stratification score, we evaluated the degree of agreement between FRS and QRS in 10-year CVR in disease-free individuals attending a teaching hospital.

## Methods

### Study design

This was a cross-sectional, observational, descriptive, analytical study.

#### Subjects

From January 2014 to January 2015, patients who attended scheduled appointments at the Internal Medicine outpatient clinic of a university hospital in the south of Brazil were invited to participate in the study. A total of 120 patients were assessed and, using a convenience sample, our final sample consisted of 74 patients.

### Data collection instruments and procedure

#### Standard form

Data were obtained by interview with participants or from their medical records by medicine students. A standard form (Appendix I) was filled out with these data, including identification, clinical, and laboratory data, as follows.

1. **Identification** - Age (years), considering geriatric (≥ 60 years) and non-geriatric age (< 60 years); sex (female or male); ethnicity (white or non-white); self-defined ethnicity (white or non-white); family income per dependent (self-reported income in minimum wages - MW, BRL724.00^7^ - divided by the number of family members), which was classified into high income (> 1MW) or low income (≤ 1 MW); educational attainment - low educational attainment (primary education or none) and high educational attainment (from 'some high school education' to 'bachelor's degree').
2. **Clinical examination** - History of CVD (left ventricular hypertrophy, angina and/or acute myocardial infarction; coronary revascularization or stent; congestive heart failure; intermittent claudication; stroke or transient ischemic attack); type 1 or type 2 diabetes mellitus (DM) (previous diagnosis or in treatment); treated systemic arterial hypertension (SAH) (previous diagnosis and/or use of anti-hypertensive drugs); history of premature coronary artery disease (CAD) (myocardial infarction or sudden death before the age of 55 of patient's father or other male first degree relative, or before the age of 65 of patient's mother or other female first degree relative);^4^ rheumatoid arthritis; atrial fibrillation; chronic kidney disease (previous diagnosis) and smoking - non-smokers or smokers (current smokers or who had quit smoking less than 2 years prior to the study).^8^ Weight (kg) and height (m) were measured using an anthropometric scale. Systolic arterial pressure (SAP) (mmHg) was measured with patient in the supine position, using an automatic, oscillatory, sphygmomanometer, after five minutes of rest. The highest SAP value between the two upper limbs was considered for analysis.^9^ Body mass index (BMI) was calculated considering overweight and obesity as BMI ≥ 25 kg/m^2^ and > 30 kg/m^2^, respectively.^10^
3. **Laboratory tests** - Lipid profile was analyzed by total cholesterol (TC) (mg/dL) and HDL cholesterol (HDL-c) (mg/dL) levels in the last 12 months. An altered lipid profile was considered as TC ≥ 240 mg/dl and/or HDL-c < 40.^3^

#### Risk stratification scores to estimate the 10-year overall CVR

##### Framingham risk score (FRS)

FRS estimates CVR based on the variables sex, age, SAP, SAH therapy, smoking, DM, HDL-c and TC. These data were used in the calculators available at Framingham Heart Study website.^11^

Individuals with a 10-year overall CVR ≥ 20% were classified as at high risk. Participants without recent data of lipid profile had their risk estimated by a calculator that used the BMI (in place of lipid data).^12^

##### QRISK-2 score (QRS)

QRS estimates CVR based on the variables gender, age, ethnicity, smoking, DM, family history of premature CAD, atrial fibrillation, SAH therapy, rheumatoid arthritis; chronic kidney disease; TC/HDL ratio and BMI. QRS was calculated using the risk calculator available at ClinRisk database website.^13^ Patients with a risk probability ≥ 20%^14^ were classified as at high risk (Table 1).

**Table 1:** Characteristics of 10-year cardiovascular risk stratification tools

**Score**	**Local/Studies on instrument derivation**	**Age**	**Gender**	**Variables**	**Outcomes**	**Risk**
FRS, CVD in 10 years; 2 versions, FRS with lipid levels and FRS with BMI	8,491 participants, Framingham, Massachusetts, United Sates of America, 12 years of follow-up	30-74 years	Male and female	Age, gender, SAP, SAH treatment, TC, HDL-c, DM, smoking, BMI	10-year risk for AMI, coronary insufficiency, angina, ischemic stroke, hemorrhagic stroke, PAOD, heart failure	0-6% low; 6-20% moderate; ≥ 20% high
QRS, CVD in 10 years	2.3 million participants, QRESEARCH database (extracted from primary health care in the United Kingdom), 5.7 years of follow-up	25-84 years	Male and female	Age, gender, ethnics, zip code, smoking, DM, angina, family history of premature CAD in first-degree relative, CKD, AF, SAH treatment, RA, TC / HDL-c ratio, SAP, BMI	10-year risk for AMI, angina, CAD, stroke and TIS	0-10% low; 10‑20% moderate; ≥ 20% high risk

FRS: Framingham risk score; QRS: QRISK-2 score; BMI: body mass index; SAP: systemic arterial pressure; SAH: systemic arterial hypertension; TC: total cholesterol; HDL-c: HDL-cholesterol; DM: Diabetes Mellitus; CAD: coronary artery disease; CKD: chronic kidney disease; AF: atrial fibrillation; RA: rheumatoid arthritis; TIS: transient ischemic stroke; PAOD: peripheral artery obstructive disease.

SOURCE: Framingham Heart Study^11^ and ClinRisk^®13^

Participants without recent lipid profile data had their risk determined by multiple imputation technique for missing data.

### Criteria definition

#### Inclusion criteria

Patients of both sex aged between 30 and 74 years (which was the widest age range common for both scores) with no evidence of CVD were included.

#### Exclusion criteria

Patients with CVD, out of the age range established by the online CVR calculators, or with missing or unreadable information in the medical records were not included in the study.

### Statistical analysis

Continuous variables with normal distribution (determined by the Kolmogorov-Smirnov test) were expressed as mean and standard deviation, whereas categorical variables as absolute frequency and proportion. Associations between variables were assessed by the chi-squared test (χ^2^), and statistically significant variables were adjusted by linear regression model, which considered the relationship between two risk strata (low/moderate *vs* high risk) as dependent variables. Models were adjusted by backward stepwise analysis and calculation of odds ratio (OR).

Kappa statistics was used to quantitatively assess the agreement between the scores. Kappa coefficients range from -1 to 1 and are classified, according to Landis & Rock (1977):^15^ < 0, no agreement; 0-19, poor agreement; 0.20-0.39, fair agreement; 0.40-0.59, moderate agreement; 0.60-0.79, substantial agreement; 0.80-0.99, almost perfect agreement; 1, perfect agreement. Statistical significance was set at p < 0.05, and analyses were performed using the SPSS® (Statistical Package for the Social Sciences), version 22.0.

### Ethical aspects

The research was approved by the Ethics Committee (project number 1973.8713.8.0000.0121), and informed consent was obtained from all participants before entering the study.

## Results

A total of 120 patients were assessed, and the final sample consisted of 74 patients (33 were excluded for CVD and 13 were out of the pre-established age range). Sociodemographic, clinical and laboratory characteristics of patients are described in Table 2. Fourteen (19%) patients did not have recent data of lipid profile.

**Table 2:** Sociodemographic, clinical and laboratory profile of the study population by continuous and categorical variables, University Hospital, Florianopolis, Brazil, 2015

**Continuous variables (n = 74)**	**Mean (**± **DP)**
Age	53.2 (± 11.0)
SAP (mmHg)	141.52 (± 24.3)
BMI (kg/m^2^)	28.4 (± 5.0)
**Continuous variables (n = 60)**	**Mean (± DP)**
CT (mg/dl) *	195.46 (± 47.7)
HDL-c*	52.0 (± 14.4)
**Continuous variables (n = 64)**	**Mean (± DP)**
Family income †	2073.1 (± 1267.1)
**Categorical variables (n = 74)**	**Total (%)**
Elderly age range	21/74 (28.3)
Female gender	48/74 (64.8)
White race	68/74 (91.8)
Low educational attainment	57/74 (77.0)
Smoking	10/74 (13.5)
Elevated SAP	38/74 (51.3)
Elevated BMI	57/74 (77.0)
**Treated SAH**	35/74 (47.2)
Female	23/48 (47.9)
Male	12/26 (46.1)
Geriatric	13/21 (61.9)
Non-geriatric	22/53 (41.5)
**Diabetes Mellitus**	18/74 (24.3)
Female	8/48 (16.7)
Male	10/26 (38.5)
Geriatric	8/21 (38.1)
Non-geriatric	10/53 (18.9)
**Categorical variables (n = 64)**	**Total (%)**
High family income †	37/64 (57.8)

* 14 patients did not have recent lipid profile data;

† 10 patients did not declare their family income. SD: standard deviation; TC: total cholesterol; HDL-c: HDL – cholesterol; SAP: systemic arterial pressure; BMI: body mass index; SAH: systemic arterial hypertension.

**Figure 1:**
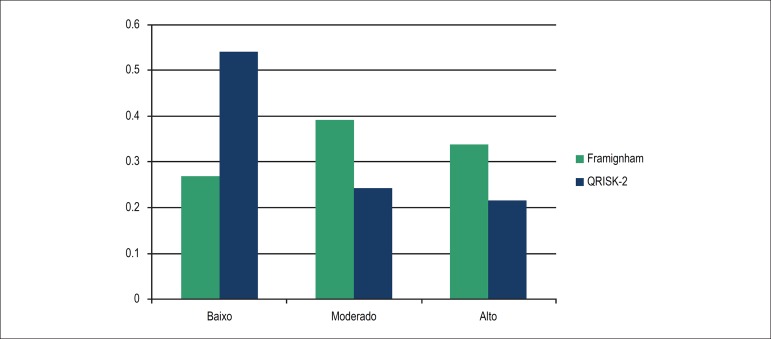
Ten-year overall cardiovascular risk stratification. University Hospital, Florianopolis, Brazil, 2015.

After risk stratification, low/moderate risk was predominant in both scores. The proportion of individuals classified as 'high risk' was higher in FRS (33.7% vs 21.6%) (Figure 1).

In both instruments, percentages of male, white individuals, non-smokers, and individuals with high SAP and BMI were higher in the high-risk stratum and similar between both scores. DM patients and older hypertensive patients were predominant in QRS and FRS, respectively, in the same stratum (Table 3).

**Table 3:** Distribution (relative and absolute frequencies) of independent variables in the high-risk stratum for cardiovascular disease, University Hospital, Florianopolis, Brazil, 2015

Variables	High risk	
	Framingham (n = 25), %[n]	QRISK-2 (n = 16), % [n]
**Age**		
Non-geriatric	48.0 [12]	31.3 [5]
Geriatric	52.0 [13]	68.6 [11]
**Gender**		
Male	64.0 [16]	62.5 [10]
Female	36.0 [9]	37.5 [6]
**Race**		
White	100 [25]	100.0 [16]
Non-white	0.0 [0]	0.0 [0]
**Treated SAH**		
No	24.0 [6]	12.5 [2]
Yes	76.0 [19]	97.5 [14]
**Family history of PCAD**		
No	72.0 [18]	62.5 [10]
Yes	28.0 [7]	37.5 [6]
**Smoking**		
No	88.0 [22]	87.5 [14]
Yes	12.0 [3]	12.5 [2]
**Diabetes Mellitus**		
No	52.0 [13]	68.8 [11]
Yes	48.0 [12]	31.3 [5]
**SAP**		
Normal	24.0 [6]	25.0 [4]
Increased	76.0 [19]	75.0 [12]
**BMI**		
Normal	16.0 [4]	18.8 [3]
Increased	84.0 [21]	81.3 [13]

CVD: cardiovascular disease; SAH: systemic arterial hypertension; PCAD: premature coronary artery disease in first-degree relative; SAP: systemic arterial pressure; BMI: body mass index.

Kappa analysis revealed moderate agreement between FRS and QRS (K = 0.519; 95%CI 0.386-0.652; p < 0.001).

In both instrument, significant relationships were observed between high-risk stratum and older age (FRS and QRS [p < 0.001 and p = 0.001]), male sex (FRS and QRS [p < 0.001 and p = 0.001]), treated SAH (FRS and QRS [p < 0.001 and p < 0.001]), DM (FRS and QRS [p < 0.001 and p < 0.001]), and elevated SAP (FRS and QRS [p = 0.002 and p < 0.003]) (Table 4). OR was higher in the presence of these variables in both FRS and QRS, with statistically significant relationship (Table 5). In both scores, DM was associated with high risk, and in QRS, high risk was strongly related with treated SAH and older age (Table 5).

**Table 4:** Bivariate analysis of independent variables in the high-risk stratum, University Hospital, Florianopolis, Brazil, 2015

Variables	Sample (%)[n = 74]	High risk			
		Framingham		QRISK-2	
		χ^2^	P†	χ^2^	p†
**Age**					
Non-geriatric	71.6 [53]	10.36	0.001	16.37	< 0.001
Geriatric	28.4 [21]				
**Gender**					
Male	35.1 [26]	13.80	< 0.001	6.70	0.01
Female	64.9 [48]				
**Race**					
White	91.9 [68]	††	††	1.80	0.18
Non-white	8.1 [6]				
**Treated SAH**					
No	52.7 [39]	12.47	< 0.001	13.23	< 0.001
Yes	47.3 [35]				
**Family history of PCAD**					
No	20.3 [15]	††	††	3.75	0.53
Yes	79.7 [59]				
**Smoking**					
No	86.5 [64]	0.74	0.78	0.18	0.89
Yes	13.5 [10]				
**Diabetes Mellitus**					
No	75.7 [56]	15.71	< 0.001	21.88	< 0.001
Yes	24.3 [18]				
**SAP**					
Normal	48.6 [36]	9.18	0.002	4.57	0.03
Increased	51.4 [38]				
**BMI**					
Normal	23.0 [17]	1.03	0.30	0.20	0.65
Increased	77.0 [57]				

* Bracketed values indicate the absolute number of participants.

† chi-squared test;

†† variables not predicted by Framingham risk score; SAH: systemic arterial hypertension; PCAD: premature coronary artery disease in first‑degree relative; SAP: systemic arterial pressure; BMI: body mass index.

**Table 5:** Independent variables and high-risk chance, University Hospital, Florianopolis, Brazil, 2015

Variables	Score					
	Framingham			Q-RISK 2		
	OR	95%CI	p*	OR	95%CI	p*
**Low/Moderate *vs*. high risk**						
Male gender	6.93	2.37-20.25	< 0.001	4.37	1.365-14.02	0.013
Geriatric age range	5.55	1.86-16.52	0.002	10.56	3.002-37.14	< 0.001
Treated SAH	6.53	2.18-19.52	0.001	12.33	2.552-59.60	0.002
Increased SAP	5.00	1.69-14.76	0.004	3.69	1.064-12.81	0.04
Diabetes mellitus	9.53	2.83-32.06	< 0.001	16.03	4.283-59.98	< 0.001

* Logistic regression analysis; OR: odds ratio; CI: confidence interval; SAH: systemic arterial hypertension; SAP: systolic arterial pressure.

Multivariate logistic regression revealed that male sex had a synergistic effect with SAH treatment in the high-risk stratus in both scores, and with older age in QRS. The variable SAP was excluded from the final model (Table 6).

**Table 6:** Multivariate analysis between risk strata and independent variables, University Hospital, Florianopolis, Brazil, 2015

Variables	Score					
	Framingham			Q-RISK 2		
	OR	IC 95%	p*	OR	IC 95%	p*
**Low/Moderate *vs*. High risk**						
Male gender	14.25	2.65-76.74	0.002	9.56	1.27-71.78	0.028
Geriatric age range	4.74	1.19-18.89	0.028	19.58	2.69-142.78	0.003
Treated SAH	10.79	1.88-61.88	0.008	19.22	1.76-210.41	0.015
**Increased SAP†**	-	-	-	-	-	-
Diabetes mellitus	3.52	0.81-15.26	0.092	9.98	1.58-62.98	0.014

* Logistic regression analysis;

† increased SAP was removed from the model; OR: odds ratio; SAH: systemic arterial hypertension; SAP: systemic arterial pressure; CI: confidence interval.

## Discussion

CVR stratification scores are constructed from population-based cohort studies, via logistic regression analysis. Risk estimates may be influenced by many factors, including calendar year, geographic region, number of visits to physician assistant office, time of patients' follow-up, quality of data collection, and prevalence of CVD risk factors. Therefore, agreement between scores, and consequently the number of individuals allocated in each risk stratum may vary substantially according to the score adopted,^14^ which was observed in this study. We found a moderate agreement (p < 0.001) between FRS and QRS in the estimate of overall 10-year CVR, which is consistent with other similar studies in the literature.^14,16^ However, a comparative study of North American and European risk scores, used in Latin American population, showed a weak agreement between scores.^17^ Thus, application of a score that had not been calibrated for the Brazilian population may lead to different therapeutic decisions.

CVR is estimated by the sum of the weights assigned to each risk factor, and multiplicative effect of these factors. Due to interaction complexity, intuitive risk estimates usually lead to underestimation or overestimation of data, and identification of these associations is made by risk scores.^3^ In the present study, FRS showed a fourteen-time higher risk for male individuals being classified as at high risk when the variable was associated with old age (OR 14.25; 95%CI 2.65-76.74; p = 0.002). QRS also showed increased probability of high risk for men (OR 9.56; 95%CI 1.27-71.78; p = 0.028) and hypertensive subjects (OR 19.22; 95%CI 1.76-210.41; p = 0.015), when associated with old age, suggesting a synergistic effect between these risk factors. The moderate agreement between the two scores used in this study may be partly due to these results, which is in agreement with the literature.^5,18^

There was an agreement between FRS and QRS algorithms in grouping men with high SAP and BMI into high risk, indicated by the higher proportion of these categories in the stratum. Relatively more importance was given to older and hypertensive individuals by the QRS, since greater proportions of these subjects (68.6% and 97.5%, respectively) were found in the high-risk stratum. As compared with the FRS, the QRS adopts a wider age range (24-84 years) for risk calculation, and assigns more weight to age, which explains the greater proportion of elderly subjects considered as at high risk by the score. Similar trend was observed for SAH, due to its direct, linear relationship with age.^9^

The highest individual contribution to high risk was given by DM (FRS and QRS [OR 9.53 and 16.03, respectively]; p < 0.001), indicating the need to identify and control this condition. However, the proportion of diabetics was higher in low/moderate risk strata, in contrast to the Adult Treatment Panel III and the Brazilian Society of Cardiology recommendations, which consider DM as a coronary disease, with high CVR.^4,19^ These recommendations are questioned by some authors of prospective studies conducted in the United Kingdom (UK), who suggest that DM should be assessed according to the age of onset and disease duration for proper risk stratification.^20^ A systematic review indicates that CVR stratification scores currently available in the literature are able to stratify the risk in these patients, differently from other instruments specific for DM, and can be used in the making decision process. However, if the physician intends to calculate the risk, as part of preventive strategy to motivate the patients to change habits and adhere to treatment, an individualized, clinical assessment of patients, without the use of instruments dominated by fixed variables may be more adequate.^21^

Bivariate analysis showed that elderly age range and male sex are associated with high risk in both scores. Aging independently contributes to the increase of CVR in every decade of life, and is related to other conditions, including obesity, treated SAH, diabetes and dyslipidemia.^22^ Male gender is an independent risk variable for coronary disease, a frequent, early manifestation of CVD, commonly reported in population studies.^12,23,24^ The association of treated SAH, increased SAP and DM with high risk is in agreement with a multi-country, case-control study that identified these factors as major contributors to attributable risk for acute myocardial infarction (AMI).^25^ These findings suggest that preventive and therapeutic strategies may be used in different populations, aiming at preventing new cases of early IAM. Also, they may serve as a base for the use of FRS and QRS, even if they had not been calibrated for the Brazilian population, as an auxiliary tool for decision making in primary health care.

The relatively higher number of patients classified as at high risk by the FRS compared with QRS (33.7% versus 21.6%) is consistent with a study conducted in the United Kingdom, which suggests that the FRS may overestimate the risk by 5% in that population.^5^ A study analyzing the use of North American instruments in the UK population revealed that the scores overestimated the results by 25-115%. This variation may be justified by the fact that these tools derived from a cohort of predominantly white men decades ago, when the prevalence of CVD in the United States was higher than today.^6^

The QRISK-2 derives from a more recent and heterogeneous cohort in the UK, in a period when the prevalence of CVD has decreased. This may explain the lower proportion of patients in the high-risk stratum identified by this score.^5^ Other factors include the use of preventive therapies (aspirin, lipid-lowering and antihypertensive drugs), which reduce the incidence of CVD and modify the quantitative relationship between risk factors and cardiovascular events in an unpredictable manner, contributing to lower agreement between the instruments.^6^

The high proportion of individuals at high risk in both scores may be attributed to characteristics of the sample, which constituted patients attending a teaching hospital that treats highly complex cases, referred from the primary health care level.

In the present study, predominance of older (71.6%), female (64.86%) patients is similar to that observed in other patient groups in this outpatient care center^26,27^ and in another Brazilian hospital.^28^ The predominance of white individuals (91.9%) is compatible with the 2010 census, conducted by the Brazilian Institute of Geography and Statistics, that reported a similar proportion in the city (94.47%).^29^

Low socioeconomic status (low income, low educational attainment and/or poor regions) is considered a psychosocial factor for CVD that negatively affects the adherence to a healthy life style, medical advice and treatment. The present study showed a predominance of patients with low educational attainment, and is consistent with another study performed in our center, showing an association between this variable and higher risk for hypertension. The higher proportion of high-income individuals may be associated with data bias due to the private nature of this question.

An Australian study^30^ related the high risk for cardiovascular disease with low socioeconomic status, suggesting the use of such association in the early detection and correct management of individuals at high risk. This strategy was successfully used in the UK, by using the QRISK-2 associated with Townsend index (which identifies each region by its zip code), and could be adapted to Brazil, in which primary healthcare centers are also strategically distributed, as in Australia and in the UK.

The elevated proportion of individuals with high SAH, SAP, overweight / obesity, and DM found in this institution reflects its position as a referral center, and corroborates the relevance of these modifiable risk factors in the study population. This may also justify the adoption of public health strategies focused on these factors.

There is as a considerable proportion of subjects without recent lipid levels (19%), which makes difficult the use of scores based on this laboratory parameter. Gaziano et al.^31^ found that the estimation of CVR by using easily obtained risk factors, such as arterial pressure, hypertension treatment, socioeconomic status, smoking, BMI and history family of DM, is able to estimate CVR as efficiently as laboratory tests, which is valuable for poorer areas. The authors also suggest the sedentary lifestyle, an important risk factor for CVD, may be included as a risk score, since it is influenced by behavior and due to the increasing prevalence of overweight/obesity worldwide. This was evidenced by a high proportion [77.0% (57/74)] of overweight/obesity detected in our study.

### Limitations, contributions and future perspectives

This study used a convenience sample, which may not reflect the behavior of the general population. Also, analysis of the effect of the combination of DM and elevated SAP with the other variables in the multivariate analysis is limited by the small sample size. The study investigated characteristics of two instruments normally used in CVR stratification in patients attending the internal medicine outpatient care of a university hospital. These data may be used as a base for the development of instruments for cardiovascular prevention, specific for local reality.

## Conclusion

In our study population, there was a moderate correlation between FRS and QRS in estimating 10-year overall CVR. The scores assign different weights to the variables, which may have a synergistic effect and be affected by local population. This finding should be recognized for clinical purposes, as well as the need for calibrating risk scores for the Brazilian population.
